# The Fever Microbe

**Published:** 1890-05

**Authors:** 


### THE FEVER MICROBE.

An illustration of a strange fact is found in the experience with the "Jamestown," now the training ship at Baltimore. On one of her trips yellow fever appeared on board, and several deaths followed. Subsequently the vessel was thoroughly renovated and extensively repaired. Her woodwork was steamed. Then she remained in northern harbors for several winters. She was finally ordered south again, and before she reached the fever district a case was developed and the patient died. Above his hammock was found a quantity of filth. The woodwork was torn out, and the filth removed. But she is still a fever ship. Then again, is the case of the "Portsmouth." She once had fever on board. Long afterward she was ordered to Norfolk for repairs. Naval Constructor Hichborn had charge of the work. A number of his workmen died, and he himself was taken down with typhoid fever, and his life was despaired of. It is true, that once a fever ship always a fever ship.

---

PROF. LOISETTE'S "Memory" Institute, 237 Fifth avenue, New York city, is pronounced a practical success. Send for a prospectus, free.

